# Genome Sequence of the Yeast *Clavispora lusitaniae* Type Strain CBS 6936

**DOI:** 10.1128/genomeA.00724-17

**Published:** 2017-08-03

**Authors:** Pascal Durrens, Christophe Klopp, Nicolas Biteau, Valérie Fitton-Ouhabi, Karine Dementhon, Isabelle Accoceberry, David J. Sherman, Thierry Noël

**Affiliations:** a Laboratoire Bordelais de Recherche en Informatique (LaBRI), UMR-CNRS 5800, Talence, France; b Université de Bordeaux, Bordeaux, France; c INRIA Joint Project Team PLEIADE, Talence, France; d Laboratoire de Microbiologie Fondamentale et Pathogénicité UMR-CNRS 5234, Bordeaux, France; e Genotoul Bioinfo, MIAT, INRA Toulouse, Castanet-Tolosan, France

## Abstract

*Clavispora lusitaniae*, an environmental saprophytic yeast belonging to the CTG clade of *Candida*, can behave occasionally as an opportunistic pathogen in humans. We report here the genome sequence of the type strain CBS 6936. Comparison with sequences of strain ATCC 42720 indicates conservation of chromosomal structure but significant nucleotide divergence.

## GENOME ANNOUNCEMENT

*Clavispora lusitaniae*, a teleomorph of *Candida lusitaniae*, is an environmentally ubiquitous ascomycetous yeast with no known specific ecological niche. It can be isolated from different substrates, such as soils, waters, plants, and gastrointestinal tracts of many animals including birds, mammals, and humans. In immunocompromised hosts, *C. lusitaniae* can be pathogenic and is responsible for about 1% of invasive candidiasis, particularly in pediatric and hematology-oncology patients (1).

So far, 2 strains have had their genomes sequenced: ATCC 42720, isolated from the blood of a patient with myeloid leukemia (2), and MTCC 1001, a self-fertile strain isolated from citrus (3). We report here the genome sequencing and assembly of the *C. lusitaniae* type strain CBS 6936 (4), isolated from citrus peel juice. Genomic DNA was isolated from a 50-mL yeast extract-peptone-dextrose culture, after spheroplast osmotic lysis and glass-rod purification of ethanol-precipitated nucleic acids. The DNA library was prepared from 1 μg according to the NEBNext DNA library prep master mix set for the Illumina (E6040) protocol with an insert size of 368 ± 122 nucleotides generated with a Covaris ultrasonicator.

The library was sequenced on the Illumina MiSeq version 1.18.54 platform, producing approximately 1.5 million paired-end reads, representing a 28× coverage. A *de novo* assembly of the reads was performed to compare the contigs to those of strain ATCC 42720. We used SPAdes version 3.9.0 with default parameters (5) to perform the assembly. Only the 53 scaffolds longer than 1 kb were retained, as the GC% of many shorter ones deviated from the average.

The scaffolds represent an overall length of 12.0 Mb, which is close to the 12.11 Mb of the ATCC 42720 genome. The GC contents amount to 44.5%, which is identical to that of strain ATCC 42720. The alignment of the CBS 6936 scaffolds against the ATCC 42720 contigs by Nucmer (6) shows almost colinearity between the two sets, apart from a 66-kb-long inversion. The number of single nucleotide polymorphisms (SNPs) between CBS 6936 and ATCC 42720 amounts to 132,141 with Phred quality higher than 30, according to Burrows–Wheeler alignment (7) of reads and FreeBayes detection (8). This SNP density of 1 SNP per 90 bp is twice the level observed between strains SC5314 and WO-1 of *Candida albicans*, which are members of different subgroups within the species and qualified as having diverged relatively recently (9).

Genes for 4 rRNAs, 197 tRNAs, and 5,539 proteins were predicted by alignment, TRNAscan-SE and a combination of Augustus (10), Snap (11), and GeneMark-ES (12), respectively. The average identity of proteins between the two strains amounts to 89.9% ± 23.2%, based on BLASTp (13) best-hit alignments.

Altogether, these results indicate that, in spite of a conserved genome structure, the sequences are different, meaning that the strains underwent a significant divergence.

### Accession number(s).

This whole-genome shotgun project has been deposited at DDBJ/EMBL/GenBank under the accession numbers SRP075809 for reads and LYUB00000000 for scaffolds. The second version of the assembly of CBS 6936 is version LYUB02000000 and is described here.
